# Review of type-2 diabetes mellitus illness perceptions and associations with illness management

**DOI:** 10.1093/geroni/igaa057.3286

**Published:** 2020-12-16

**Authors:** Samuel Hatala, Cleveland Shields

**Affiliations:** Purdue University, West Lafayette, Indiana, United States

## Abstract

Objective: Determine illness perceptions associated most frequently with measures of type-2 diabetes mellitus (T2DM) maintenance. We measured illness perceptions using the Illness Perception Questionnaire (IPQ) and variants (IPQ-Revised and Brief IPQ) Design: Review of literature from publication of IPQ to September 2020. Searched for articles utilizing IPQ but no other models of illness perception and studying T2DM Main Outcome Measures: Glucose control (measured by HbA1c levels), adherence to medications, and adherence to diet, exercise, and other lifestyle recommendations Results: Symptom attribution and fear of consequences are frequently associated with worse T2DM management and sense of control of illness progression and positive emotional valence are frequently associated with better T2DM management. Other subscales have less frequent but generally positive associations with the exceptions of recurring thoughts about T2DM duration, which had a negative association with management, and understanding the causes of T2DM, which had no associations at all. Other reviews found similar associations and highlighted a need for more general T2DM education. Conclusion: Future T2DM management interventions should promote sense of control over illness progression and positive emotional valence and provide education regarding symptoms to expect. Interventions should also consider managing patient fear, which has many associations with worse management.

